# Differential pattern of *RP1* mutations in retinitis pigmentosa

**Published:** 2010-07-15

**Authors:** Xin Zhang, Li Jia Chen, Jonathan P. Law, Timothy Y.Y. Lai, Sylvia W.Y. Chiang, Pancy O.S. Tam, Kwan Yi Chu, Ningli Wang, Mingzhi Zhang, Chi Pui Pang

**Affiliations:** 1 Department of Ophthalmology and Visual Sciences, the Chinese University of Hong Kong, Hong Kong, China; 2 Beijing Tongren Hospital, Capital Medical University, Beijing, China; 3 Joint Shantou International Eye Center, Shantou University Medical College, Shantou, China

## Abstract

**Purpose:**

Retinitis pigmentosa 1 (*RP1)* is a major gene responsible for both autosomal dominant and autosomal recessive retinitis pigmentosa (RP). We have previously identified three disease-causing mutations out of 174 RP patients. In this study, we investigated a new cohort of Chinese RP patients to further evaluate the contribution of *RP1* mutations to cause RP.

**Methods:**

A group of 55 nonsyndromic RP patients, the majority of them isolated cases or without information on family history, were screened for mutations in the entire coding sequences of *RP1*, using direct DNA sequencing. All detected variants were genotyped in 190 controls, while the three putative mutations were additionally genotyped in 362 controls subjects. Web-based programs, including PolyPhen, Sorting Intolerant from Tolerant (SIFT), Prediction of Pathological Mutations (PMUT), Single Amino Acid Polymorphism Disease-Association Predictor (SAP), ScanProsite, and ClustalW2, were used to predict the potential functional and structural impacts of the missense variants on RP1.

**Results:**

A total of 14 sequence changes were identified. Among them, five were novel and found only in the RP patients. Two missense variants (p.K1370E and p.R1652L), which are conserved in primates, were predicted to have functional and structural impacts on the RP1 protein. The other three variants (c.787+34T>C, p.I408L and p.L2015L) were considered benign.

**Conclusions:**

If these two novel missense variants are in fact pathogenic, then *RP1* mutations account for approximately 2.18% (5/229) of RP cases in our Chinese cohort; this is similar to other ethnic groups. However, a relatively higher frequency of missense mutations found in the Chinese patients may suggest an ethnic diversity in the *RP1* mutation patterns.

## Introduction

Retinitis pigmentosa (RP; OMIM 268000; Mendelian Inheritance in Man; National Center for Biotechnology Information, Bethesda, MD) refers to a heterogeneous group of inherited retinal dystrophies characterized by night blindness and progressive loss of visual field due to the degeneration of rod photoreceptors in the retina. In the late stage, the central vision will be affected, resulting in irreversible blindness [1]. Prevalence of RP is between 1/4,000 [2-5] and 1/6,000 [6] in western countries. Among Chinese, the prevalence was 1/3,784 in a dispersed region of China [7]. In the Peking eye study, the estimated prevalence was as high as 1/1,000 among people aged over 40 in northern China [8].

RP can present as autosomal recessive (arRP, 10%–45%), autosomal dominant (adRP, 10%–30%), or X-linked (xlRP, 0%–15%). Around 35%–50% of RP cases have been denoted as simplex RP (sRP) because of the absence of known family history of RP or segregation pattern [2,3,5,6,9-11]. More than 40 genes have been identified to cause RP, of which 20, 26, and two genes were responsible for adRP, arRP, and xlRP, respectively (RetNet, the Retinal Information Network, provided in the public domain by the University of Texas Houston Health Science Center, Houston, TX, accessed March 7, 2010). The *RP1* locus was mapped by linkage testing in a large adRP family in southeastern Kentucky [12,13]. The gene subsequently linked to adRP was named retinitis pigmentosa 1 (*RP1*, OMIM 603937) [13]. Later studies revealed that mutations in *RP1* cause both dominant [14-17] and recessive forms of RP [18,19]. Most *RP1* mutations are single nucleotide substitutions that produce a premature stop codon or insertion/deletion changes, resulting in a truncated protein in Caucasian populations [20]. These mutations were predicted to truncate the RP1 protein by approximately 50% to 70% of the full length. Only a few missense variants, such as p.T373I [18], p.K663N [16], p.A669T [18], p.D984G [21], and p.L1808P [16], have been found to be disease causing. RP1 is expressed prominently, if not only, in the photoreceptor cells of the retina [15,19,22,23] and is involved in the correct orientation and higher order stacking of outer segment discs [24]. RP1 is a microtubule-associated protein forming part of the photoreceptor axoneme [25] and thus plays an important role in photoreceptor function.

We previously evaluated the mutation profiles of *RP1* in Chinese RP patients by studying unrelated RP patients with mixed phenotypes. We found that *RP1* mutations (p.R677X and p.D984G) accounted for about 1.2% of overall RP [21,26,27], apparently lower than that in other populations [1,28]. Recently, we identified two truncation mutations causing arRP in a Chinese family [29]. In this study, we screened for additional RP patients and control subjects to complement the mutation profile of *RP1* in Chinese patients.

## Methods

### Study subjects

All study subjects recruited were of Chinese ethnicity and were given complete ocular examinations, including slit-lamp biomicroscopy, electroretinography, and fundus photography. Diagnosis of nonsyndromic RP was based on ophthalmic investigations and absence of systemic signs associated with syndromic RP. Subjects were confirmed to be free of any other major eye diseases, except mild senile cataract in some elderly subjects. In total, 55 unrelated RP patients were recruited, including 32 females and 23 males, with ages ranging from 19 to 84 years. Inheritance patterns of the disease were defined according to the family history of the subjects. In some cases, however, there was no information about the family structure for heredity classification. The control subjects were all unrelated, older than 60 years, and had no family history of RP from which 190 of them (53 males and 137 females) were from Hong Kong and had been described in our previous studies [26,27]. We also added another 362 controls for validation of the putative mutations: 35 subjects from Hong Kong (11 males and 24 females, age range 70 to 90 years), 180 from Beijing in northern China [30] (88 males and 92 females, age range 60 to 89 years), and 147 from Shantou in southern China (53 males and 94 females, age range 63 to 96 years). All controls received routine follow-up and complete ophthalmic examination to ensure that they remained free of RP or other major eye diseases except mild senile cataract. Written informed consents were obtained from all study subjects after explanation of the nature of the study. All procedures were conducted in accordance with the tenets of the Declaration of Helsinki. The study protocol was approved by the Ethics Committee on Human Research, the Chinese University of Hong Kong and the respective review board on human subject research at each participating academic institution. Peripheral venous whole blood, 5 ml, was collected from each patient in EDTA tubes and stored at −80 °C before genetic analysis.

### Mutational screening of the *RP1* gene in cases and controls

Genomic DNA was extracted from whole blood using a commercial kit (QIAamp DNA Blood kit; Qiagen, Valencia, CA), according to the manufacturer’s instructions. In brief the frozen whole blood samples were thawed and blood cells lysed in QIAGEN Protease and Buffer AL in the kit. The DNA was obtained by precipitation. Twenty-six amplicons covering all three coding exons and the exon–intron boundaries of *RP1*, as previously described [21,26], were amplified in all patients and the 190 Hong Kong controls by PCR and analyzed by direct DNA sequencing using dye-termination chemistry (Big-Dye Terminator Cycle Sequencing Reaction Kit; ver. 3.1; Applied Biosystems, Inc., Foster City, CA) on a DNA sequencer (3130XL; Applied Biosystems), according to the supplier’s protocol. The DNA sequences were compared with the human *RP1* (ENSG00000104237) sequence in the Ensembl database. Any rare variants detected were double confirmed by bidirectional sequencing. The novel putative mutations were further genotyped in the additional 362 control subjects.

### Analysis of variants

We defined a variant as “novel” if it had not been reported in the literature or registered in the Single Nucleotide Polymorphism (SNP) database (provided in the public domain by the National Center for Biotechnology Information, Bethesda, MD). A variant was regarded as potentially disease causing if it was (1) expected to alter the amino acid sequence of the protein, (2) exclusively observed in patients with RP, while completely absent from the control subjects, and (3) predicted to alter the protein structure or function through in silico analysis.

For the common *RP1* sequence variants with minor allele frequency >5% detected in both patients and controls, genotype frequencies were compared between the two groups in a dominant genetic model using the χ^2^ test in SPSS (ver.15.0; SPSS Inc., Chicago, IL). A Bonferroni method was used to correct the p values in multiple comparisons. For the rare missense variants detected exclusively in the RP patients, we used four web-based programs to evaluate possible biologic effects of the amino acid substitution on the structure and function of the RP1 protein, including (1) Polymorphism Phenotyping (PolyPhen provided by the Bork Group and the Sunyaev Lab, Brigham and Women’s Hospital, Harvard Medical School, Boston, MA), (2) Sorting Intolerant from Tolerant (SIFT, provided in the public domain by the J. Craig Venter Institute, Rockville, MD), (3) Prediction of Pathological Mutations (PMUT, provided by the Molecular Recognition and Bioinformatics Group, Institute for Research in Biomedicine-Barcelona Science Park and University of Barcelona, Barcelona, Spain), and (4) the Single Amino Acid Polymorphism Disease-Association Predictor (SAP disease-association predictor, provided by the Sapred Team, Center for Bioinformatics, Peking University, Beijing, China). Possible alterations of protein motifs and functional sites were assessed by scanning through the PROSITE database (ScanProsite, provided by the Swiss-Prot group, Swiss Institute of Bioinformatics, Lausanne, Switzerland). We further examined evolutionary conservation of the amino acid residues in these variants by multiple sequence alignment using the ClustalW2 program (provided by the European Molecular Biology Laboratory, European Bioinformatics Institute, Cambridge, UK).

## Results

### Classification of the retinitis pigmentosa cases

Based on family information, the 55 nonsyndromic RP patients presented with mixed inheritance patterns, with five (9.09%) being classified as adRP, five (9.09%) as arRP, two (3.6%) as xlRP, and 23 (41.8%) as sRP. The remaining 20 patients had an unknown inheritance pattern due to the lack of familial data.

### Sequence variants detected in the *RP1* gene and pathogenicity assessment

A total of 14 *RP1* variants were identified (Table 1). Among them, six (p.R872H, p.N985Y, p.A1670T, p.S1691P, p.Q1725Q, and p.C2033Y) were common SNPs registered in the SNP database. These six SNPs were found at high frequencies among the control subjects. Association analyses showed that none of them was significantly associated with RP (p_corr_>0.05). Therefore, they were not considered disease-causing mutations.

**Table 1:** Sequence variants detected in the *RP1* gene among 55 Chinese RP patients.

				**Variation frequency +**	
**Location**	**Nucleotide change**	**Residual change**	**Description**	**Patients** **(n=55)**	**Controls** **(n=190)**
Intron 3	c.787+34T>C	/	Novel	20090	0/190
Exon 4	c.1222A>C	p.I408L	Novel	20090	0/190
Exon 4	c.2116G>C *	p.G706R	[26]	20121	1/190
Exon 4	c.2615G>A	p.R872H	rs444772	43/55	130/190
Exon 4	c.2953A>T	p.N985Y	rs2293869	20149	27/190
Exon 4	c.3024G>A	p.Q1008Q	[26]	20090	1/190
Exon 4	c.4108A>G	p.K1370E	Novel	20090	0/190
Exon 4	c.4955G>T	p.R1652L	Novel	20090	0/190
Exon 4	c.5008G>A	p.A1670T	rs446227	44/55	106/190
Exon 4	c.5071T>C	p.S1691P	rs414352	44/55	106/190
Exon 4	c.5175A>G	p.Q1725Q	rs441800	44/55	106/190
Exon 4	c.6045A>G	p.L2015L	Novel	20090	0/190
Exon 4	c.6098G>A	p.C2033Y	rs61739567	20149	27/190
Exon 4 (3′UTR)	c.6542C>T	/	[26]	20149	2/190

*One patient was homozygous for the p.G706R variant while the other was heterozygous for the variant; +The variation frequency was presented in a format of “variant genotypes/total genotypic counts”; / indicates “not applicable.”

We also identified eight rare variants, three of which (p.G706R, p.Q1008Q, and c.6542C>T) occurred in both the case and control subjects. The remaining five were novel and not detected in the 552 control subjects from different areas of China. Two of them (p.L2015L and intronic c.787+34T>C) did not lead to amino acid change, while the other three (p.I408L, p.K1370E, and p.R1652L) were missense changes.

**Figure 1:**
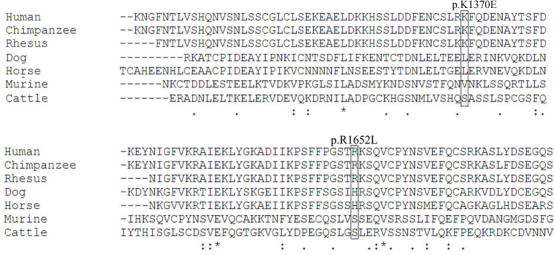
Multiple protein sequence alignment of retinitis pigmentosa 1 (*RP1*; partially), showing the location of p.K1370E and p.R1652L. The two residues are not conserved across different species. The accession numbers of the RP1 protein sequences of different species are as follows: Human NP_006260.1, chimpanzee (*Pan troglodytes)* XP_528138.2, rhesus monkey (*Macaca mulatt*a) XP_001083644.1, dog (*Canis lupus)* NP_001003040.1, horse (*Equus caballus)* XP_001498071.2, murine (*Mus musculus)* NP_035413.1, and cattle (*Bos taurus)* NP_776383.1

The impact of variant p.I408L was predicted to be “Borderline” by PolyPhen, “Tolerated” by SIFT, and “Neutral” by PMUT, with a maximum reliability index of 9 (Table 2). The Grantham score was 5. Based on these assessments, p.I408L is less likely to be a disease-causing mutation. The variant p.K1370E was predicted as “Potentially damaging” and “Potentially Intolerant” by PolyPhen and SIFT, respectively. Although predicted to be “Pathological” by PMUT (reliability index of 0). The Grantham score for this substitution was 56, being moderately conservative. Therefore, p.K1370E is potentially disease causing. For the p.R1652L variant, the predictions were “Possibly damaging” by PolyPhen, “Potentially Intolerant” by SIFT, and “Pathological” (reliability index of 7) by PMUT. With a Grantham score of 102, p.R1652L should be moderately radical and highly likely to be a functional mutation. PROSITE scanning showed that no structural or functional domain was present at or around these two residues. However, the variant p.R1652L was predicted to abolish one possible protein kinase C phosphorylation site on the RP1 protein (Table 3). Determination of the evolutionary conservation of p.K1370E and p.R1652L by multiple amino acid sequence alignment of the human RP1 with the protein sequences derived from chimpanzee, rhesus monkey, dog, horse, murine, and cattle showed that both residues 1370 and 1652 in RP1 were conserved among the primates (Figure 1).

**Table 2:** Carriers and pathogenic potentials of the 3 novel *RP1* nonsynonymous variants.

	**Nucleotide**	**Residual**	**Case**				**PolyPhen**		**SIFT**		**PMUT**		**Grantham**
**Location**	**change**	**change**	**Type**	**Sex**	**Age**	**Control**	**Prediction**	**PSIC score**	**Prediction**	**Score**	**Prediction**	**RI**	**Score**
Exon 4	c.1222A>C	p.I408L	adRP	F	56	0/552	Borderline	1.234	Tolerant	0.29	Neutral	9	5
Exon 4	c.4108A>G	p.K1370E	sRP	F	61	0/552	Potentially damaging	1.477	Potentially Intolerant	0.09	Pathological	0	56
Exon 4	c.4955G>T	p.R1652L	sRP	F	50	0/552	Possibly damaging	1.63	Potentially Intolerant	0.1	Pathological	7	102

Type: Type of RP; PSIC: Position-Specific Independent Counts, a scoring system used in PolyPhen; RI: Reliability Index.

**Table 3:** Protein domain and post-translational modification site predictions for the RP1 wild type protein and the missense variants, p.K1370E and p.R1652L, were analyzed by web-based analysis program ScanProsite.

**A. Description**	**Wild type**	**p.K1370E**
Structural or functional domains	Nil	Nil
Post-translational modification site	Nil	Nil
**B. Description**	**Wild type**	**p.R1652L**
Structural or functional domains	Nil	Nil
Post-translational modification site		
*Residue 1650–1652*	PKC-phospho site (StR)	Nil
*Residue 1651–1653*	PKC-phospho site (TrK)	PKC-phospho site (TIK)

Difference between wild type and the p.K1370E variant, at position around residue 1370. B. Difference between wild type and the p.R1652L variant, at position around residue 1652. Nil represents None of the protein domain or post-translational modification site was predicted in related location . *PKC-phospho site: Protein kinase C phophosphorylation site. The prediction is based on the amino acid sequence pattern of a serine (S) or threonine (T) residue found close to a C-terminal basic residue such as arginine (R) or Lysine (K) [37,38].

## Discussion

**Figure 2:**
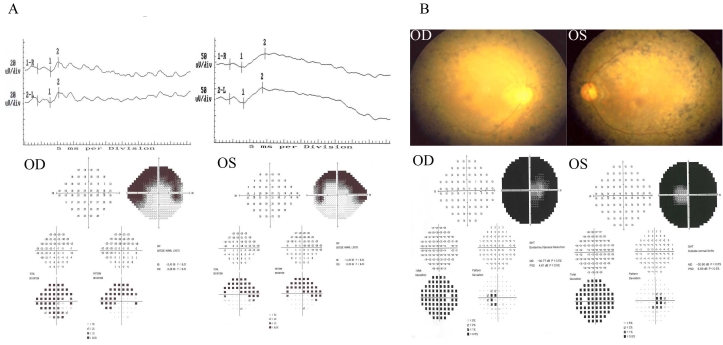
Typical RP clinical presentations of the two putative missense mutation (p.K1370E and p.R1652L) carriers. They both appear to be less severe than the truncation mutation carrier reported previously [29]. **A**: Electroretinogram (ERG) and automated visual field reports of the p.K1370E carrier. **B**: Fundus photographs and automated visual field reports of the p.R1652L carrier, showing much milder clinical manifestation in comparison to the carrier with p.S2RfxX16 and p.P1648SfsX13 truncation mutations [29]. Abbreviations: OD represents Right eye; OS represents Left eye.

In the present study, we sequenced the *RP1* gene in 55 newly recruited Chinese RP patients and identified five novel variants, providing new information about the variation pattern of the gene. Of the five variants, three missense variants were exclusively found in RP patients and absent in the 552 control subjects. Therefore, they are considered excellent candidate disease-causing mutations. Unfortunately, as the family members of these three patients did not provide consent to join our study, we were not able to perform segregation analysis for the putative mutations. We therefore used in silico analysis to provide some insights into their impacts. The three programs, i.e., PolyPhen, SIFT, and the Grantham score, are frequently used algorithms in the prediction of the functionality of missense SNPs [31,32]. However, their predictions may sometimes be inconsistent, although strong concordances have been observed [33]. We therefore applied the PMUT program to provide additional assessment. By using these tools we would expect that p.I408L is less likely to be a functional mutation (Table 2), although a segregation study would be of great interest to confirm its role. This variant occurred in a patient with adRP. Ocular and electroretinography examinations revealed a typical sign of RP (data not shown). By mutational analyses of rhodopsin (*RHO*, OMIM 180380), photoreceptor-specific nuclear receptor (*NR2E3*, OMIM 604485), and neural retina leucine zipper (*NRL*, OMIM 162080) [29], we did not find any variants in this subject (data not shown). Therefore, the adRP in this patient is likely to be caused by another yet-to-be-identified mutation. In contrast, the variant p.R1652L was likely to be a deleterious substitution with consistent predictions by PolyPhen, SIFT, PMUT, and Grantham score. Variant p.K1370E was also predicted to be deleterious by the four programs, although with a lower reliability index (Table 2). Meanwhile p.R1652 and p.K1370 were conserved among primates (Figure 1). They are located in the region of the RP1 polypeptide where many mutations occur. Furthermore, p.R1652L was predicted to abolish one possible protein kinase C phosphorylation site on the protein (Table 3). Protein phosphorylation is an important posttranslational modification process that can alter the conformation and thus function and localization of the protein [34]. The predicted abolishment of the phosphorylation site can affect the normal functioning of RP1. As a whole, our results are consistent for pathologic roles of p.R1652L and p.K1370E in RP. The patients with these postulated mutations had typical clinical presentations of RP (Figure 2), although the clinical presentations appear less severe than the truncation mutation carrier [29]. Notably, the p.R1652L and p.K1370E carriers were classified as sRP because their parents did not have the disease. Thus, these two variants could be de novo mutations causing adRP or could cause arRP in a digenic pattern co-dominantly with another yet-to-identify mutation, which is worth further investigation.

In our previous studies, we identified two *RP1* mutations, p.R677X and p.D984G, in two out of 173 Hong Kong Chinese RP patients [21,26]. Recently we identified two truncation mutations (c.5_6delGT and c.4941_4942insT) causing arRP in a Chinese pedigree [29]. Combining these findings with the two missense mutations detected in the current study, the total contribution of *RP1* mutations is approximately 2.18% (5/229, 95% confidence interval 0.93%–5.0%) in our Chinese cohort. So far, studies evaluating the *RP1* in other Chinese RP cohorts are limited. Recently, Sheng et al. [30] identified the p.N985Y variant as the disease-causing mutation for adRP, being co-segregated with the disease in the pedigree and absent in 105 controls. Interestingly, the p.N985Y variant was found to be very common in our study cohort, presenting in 14.2% (27/190) of control subjects. We do not know whether the pedigree is of Han Chinese ethnicity due to the lack of data in the Sheng et al. [30] report. We would expect, however, that ethnic diversity of the *RP1* mutation pattern also exists within Chinese populations. In the study of Sheng et al., [30] another three missense variants (p.P63I, p.G79E, and p.P903L) were also found to occur at high frequencies and exclusively in the patient group, although the disease-causative properties remained inconclusive [30]. In contrast, no truncation mutation was found in their cohort. In light of their findings and ours, missense *RP1* mutations may be playing a prominent role in the genetic epidemiology of RP in Chinese populations. Further studies in other Chinese cohorts with a larger sample size are warranted to confirm this.

**Figure 3:**
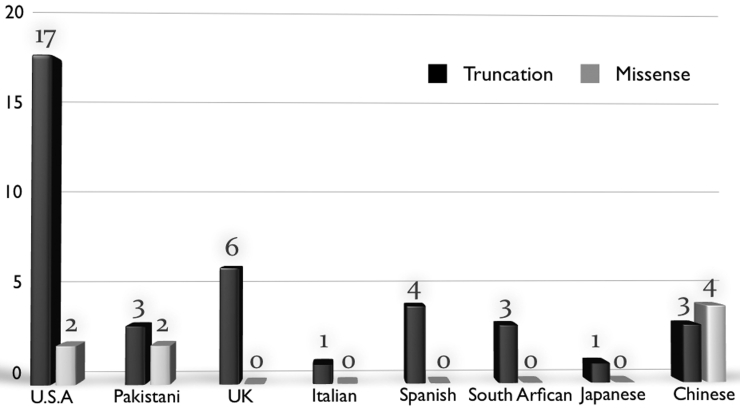
Summary of the Retinitis Pigmentosa 1 (*RP1*) mutation pattern in patients with retinitis pigmentosa among different ethnic populations. Most *RP1* mutations are single nucleotide substitutions producing a premature stop codon or insertion/deletion changes resulting in a truncated protein. Only a few missense variants have been found to be disease causing. So far only in Chinese missense variants have been found to be more than truncated mutations.

To date, at least 41 *RP1* mutations that are causative for RP have been reported, most of which are truncation mutations. It is noteworthy that, at least one-half of the known missense mutations occur in Chinese RP patients (Table 4), while only three out of 33 (9%) known truncations have been identified in all the published Chinese *RP1* mutations (Figure 3). In contrast, most known truncation mutations were detected in other populations, with the most frequent mutation, p.R677X, presenting in approximately 3% of adRP patients in the United States [15]; it has also presented at relatively high frequencies in the UK [20] and Italian [35] populations. Hence, there are distinctly differential *RP1* mutation patterns existing, if not globally, at least between the Chinese and Caucasian populations.

**Table 4:** Summary of RP1 missense mutations identified in patients with retinitis pigmentosa.

**Mutation**	**cDNA**	**Study population**	**Diagnosis**	**Reference**
p.T373I*	c.1118C>T	Pakistan	ARRP	[18]
p.K663N	c.1989G.>T	U.S.A.	ADRP	[16]
p.A669T	c.2005G>A	Pakistan	ARRP	[18]
p.D984G	c.2951A>G	Chinese	ADRP	[21]
p.N985Y	c.2954A>T	Chinese	ADRP	[39]
p.K1370E	c.4108A>G	Chinese	SRP	Present Study
p.R1652L	c.4955G>T	Chinese	SRP	Present Study
p.L1808P	c.5423T>C	U.S.A.	ADRP	[16]

Asterisk represents homozygote.

So far, most confirmed pathologic mutations in *RP1* among different populations are nonsense or frameshift mutations located in exon 4. These mutant transcripts are predicted to be insensitive to the nonsense-mediated decay pathway, leading to the production of truncated proteins lacking one-half to two-thirds of the C-terminal portion [36]. As it has been suggested that haploinsufficiency of RP1 is not causative for RP [29], these truncated proteins may exert their role in the pathogenesis of RP through a dominant negative effect. Likewise, the missense mutations may also play a role in RP etiology through a similar mechanism, which has yet to be elucidated by functional assays.

In summary, we have identified two novel missense mutations (p.R1652L and p.K1370E). These mutations were predicted to have functional impacts on RP1 and are likely to be pathogenic. In light of these findings along with the findings of our previous studies, both *RP1* truncation and missense mutations may contribute to the etiology of RP, accounting for approximately 2.18% of RP cases in our Chinese cohort, similar to other populations. Nevertheless, the Chinese population has a disproportionally high frequency of missense mutations, providing further insights into the ethnic diversity of the genetic epidemiology of RP.

## References

[1] Hartong DT Berson EL Dryja TP Retinitis pigmentosa. Lancet 2006 368 1795 809 17113430 10.1016/S0140-6736(06)69740-7

[2] Bundey S Crews SJ A study of retinitis pigmentosa in the City of Birmingham. I Prevalence. J Med Genet 1984 21 417 20 6512829 10.1136/jmg.21.6.417 PMC1049340

[3] Bunker CH Berson EL Bromley WC Hayes RP Roderick TH Prevalence of retinitis pigmentosa in Maine. Am J Ophthalmol 1984 97 357 65 6702974 10.1016/0002-9394(84)90636-6

[4] Grøndahl J Estimation of prognosis and prevalence of retinitis pigmentosa and Usher syndrome in Norway. Clin Genet 1987 31 255 64 3594933 10.1111/j.1399-0004.1987.tb02804.x

[5] Haim M Epidemiology of retinitis pigmentosa in Denmark. Acta Ophthalmol Scand Suppl 2002 233 1 34 11921605 10.1046/j.1395-3907.2002.00001.x

[6] Peterlin B Canki-Klain N Morela V Stirn B Rainer S Cerar V Prevalence of retinitis pigmentosa in Slovenia. Clin Genet 1992 42 122 3 1395082 10.1111/j.1399-0004.1992.tb03222.x

[7] Hu DN Prevalence and mode of inheritance of major genetic eye diseases in China. J Med Genet 1987 24 584 8 3500313 10.1136/jmg.24.10.584 PMC1050283

[8] Xu L Hu L Ma K Li J Jonas JB Prevalence of retinitis pigmentosa in urban and rural adult Chinese: The Beijing Eye Study. Eur J Ophthalmol 2006 16 865 6 17191195 10.1177/112067210601600614

[9] Fujiki K Hayakawa M Kanai A Matsumura M Koizumi H Tamai M Shiono T Tokoro T Akazawa Y Kubota N An epidemiogenetic study of typical retinitis pigmentosa in Japan–a preliminary report of nationwide, multicenter study. Nippon Ganka Gakkai Zasshi 1992 96 225 30 1558019

[10] Hu DN Genetic aspects of retinitis pigmentosa in China. Am J Med Genet 1982 12 51 6 7091196 10.1002/ajmg.1320120107

[11] Nájera C Millan JM Beneyto M Prieto F Epidemiology of retinitis pigmentosa in the Valencian community (Spain). Genet Epidemiol 1995 12 37 46 7713399 10.1002/gepi.1370120105

[12] Field LL Heckenlively JR Sparkes RS Garcia CA Farson C Zedalis D Sparkes MC Crist M Tideman S Spence MA Linkage analysis of five pedigrees affected with typical autosomal dominant retinitis pigmentosa. J Med Genet 1982 19 266 70 7120314 10.1136/jmg.19.4.266 PMC1048891

[13] Blanton SH Heckenlively JR Cottingham AW Friedman J Sadler LA Wagner M Friedman LH Daiger SP Linkage mapping of autosomal dominant retinitis pigmentosa (RP1) to the pericentric region of human chromosome 8. Genomics 1991 11 857 69 1783394 10.1016/0888-7543(91)90008-3

[14] Jacobson SG Cideciyan AV Iannaccone A Weleber RG Fishman GA Maguire AM Affatigato LM Bennett J Pierce EA Danciger M Farber DB Stone EM Disease expression of RP1 mutations causing autosomal dominant retinitis pigmentosa. Invest Ophthalmol Vis Sci 2000 41 1898 908 10845615

[15] Pierce EA Quinn T Meehan T McGee TL Berson EL Dryja TP Mutations in a gene encoding a new oxygen-regulated photoreceptor protein cause dominant retinitis pigmentosa. Nat Genet 1999 22 248 54 10391211 10.1038/10305

[16] Bowne SJ Daiger SP Hims MM Sohocki MM Malone KA McKie AB Heckenlively JR Birch DG Inglehearn CF Bhattacharya SS Bird A Sullivan LS Mutations in the RP1 gene causing autosomal dominant retinitis pigmentosa. Hum Mol Genet 1999 8 2121 8 10484783 10.1093/hmg/8.11.2121 PMC2585827

[17] Kawamura M Wada Y Noda Y Itabashi T Ogawa S Sato H Tanaka K Ishibashi T Tamai M Novel 2336–2337delCT mutation in RP1 gene in a Japanese family with autosomal dominant retinitis pigmentosa. Am J Ophthalmol 2004 137 1137 9 15183808 10.1016/j.ajo.2003.12.037

[18] Khaliq S Abid A Ismail M Hameed A Mohyuddin A Lall P Aziz A Anwar K Mehdi SQ Novel association of RP1 gene mutations with autosomal recessive retinitis pigmentosa. J Med Genet 2005 42 436 8 15863674 10.1136/jmg.2004.024281 PMC1736063

[19] Riazuddin SA Zulfiqar F Zhang Q Sergeev YV Qazi ZA Husnain T Caruso R Riazuddin S Sieving PA Hejtmancik JF Autosomal recessive retinitis pigmentosa is associated with mutations in RP1 in three consanguineous Pakistani families. Invest Ophthalmol Vis Sci 2005 46 2264 70 15980210 10.1167/iovs.04-1280

[20] Payne A Vithana E Khaliq S Hameed A Deller J Abu-Safieh L Kermani S Leroy BP Mehdi SQ Moore AT Bird AC Bhattacharya SS RP1 protein truncating mutations predominate at the RP1 adRP locus. Invest Ophthalmol Vis Sci 2000 41 4069 73 11095597

[21] Chiang SW Wang DY Chan WM Tam PO Chong KK Lam DS Pang CP A novel missense RP1 mutation in retinitis pigmentosa. Eye (Lond) 2006 20 602 5 15933747 10.1038/sj.eye.6701944

[22] Guillonneau X Piriev NI Danciger M Kozak CA Cideciyan AV Jacobson SG Farber DB A nonsense mutation in a novel gene is associated with retinitis pigmentosa in a family linked to the RP1 locus. Hum Mol Genet 1999 8 1541 6 10401003 10.1093/hmg/8.8.1541

[23] Sullivan LS Heckenlively JR Bowne SJ Zuo J Hide WA Gal A Denton M Inglehearn CF Blanton SH Daiger SP Mutations in a novel retina-specific gene cause autosomal dominant retinitis pigmentosa. Nat Genet 1999 22 255 9 10391212 10.1038/10314 PMC2582380

[24] Liu Q Lyubarsky A Skalet JH Pugh EN Jr Pierce EA RP1 is required for the correct stacking of outer segment discs. Invest Ophthalmol Vis Sci 2003 44 4171 83 14507858 10.1167/iovs.03-0410 PMC1904498

[25] Liu Q Zuo J Pierce EA The retinitis pigmentosa 1 protein is a photoreceptor microtubule-associated protein. J Neurosci 2004 24 6427 36 15269252 10.1523/JNEUROSCI.1335-04.2004 PMC1904502

[26] Baum L Chan WM Yeung KY Lam DS Kwok AK Pang CP RP1 in Chinese: Eight novel variants and evidence that truncation of the extreme C-terminal does not cause retinitis pigmentosa. Hum Mutat 2001 17 436 11317367 10.1002/humu.1127

[27] Chan WM Yeung KY Pang CP Baum L Lau TC Kwok AK Lam DS Rhodopsin mutations in Chinese patients with retinitis pigmentosa. Br J Ophthalmol 2001 85 1046 8 11520753 10.1136/bjo.85.9.1046 PMC1724134

[28] Roberts L Bartmann L Ramesar R Greenberg J Novel variants in the hotspot region of RP1 in South African patients with retinitis pigmentosa. Mol Vis 2006 12 177 83 16568030

[29] Chen LJ Lai TY Tam PO Chiang SW Zhang X Lam S Lai RY Lam DS Pang CP Compound heterozygosity of two novel truncation mutations in RP1 causing autosomal recessive retinitis pigmentosa. Invest Ophthalmol Vis Sci 2010 51 2236 42 19933189 10.1167/iovs.09-4437

[30] Yang Y Zhang X Chen LJ Chiang SW Tam PO Lai TY Chan CK Wang N Lam DS Pang CP Association of R2E3 but not NRL mutations with retinitis pigmentosa in the Chinese population. Invest Ophthalmol Vis Sci 2010 51 2229 35 19933183 10.1167/iovs.09-4299

[31] Li WH Wu CI Luo CC Nonrandomness of point mutation as reflected in nucleotide substitutions in pseudogenes and its evolutionary implications. J Mol Evol 1984 21 58 71 6442359 10.1007/BF02100628

[32] Mooney S Bioinformatics approaches and resources for single nucleotide polymorphism functional analysis. Brief Bioinform 2005 6 44 56 15826356 10.1093/bib/6.1.44

[33] Rudd MF Williams RD Webb EL Schmidt S Sellick GS Houlston RS The predicted impact of coding single nucleotide polymorphisms database. Cancer Epidemiol Biomarkers Prev 2005 14 2598 604 16284384 10.1158/1055-9965.EPI-05-0469

[34] Johnson LN Lewis RJ Structural basis for control by phosphorylation. Chem Rev 2001 101 2209 42 11749371 10.1021/cr000225s

[35] Ziviello C Simonelli F Testa F Anastasi M Marzoli SB Falsini B Ghiglione D Macaluso C Manitto MP Garrè C Ciccodicola A Rinaldi E Banfi S Molecular genetics of autosomal dominant retinitis pigmentosa (ADRP): a comprehensive study of 43 Italian families. J Med Genet 2005 42 e47 15994872 10.1136/jmg.2005.031682 PMC1736108

[36] Zhang J Sun X Qian Y LaDuca JP Maquat LE At least one intron is required for the nonsense-mediated decay of triosephosphate isomerase mRNA: a possible link between nuclear splicing and cytoplasmic translation. Mol Cell Biol 1998 18 5272 83 9710612 10.1128/mcb.18.9.5272 PMC109113

[37] Kishimoto A Nishiyama K Nakanishi H Uratsuji Y Nomura H Takeyama Y Nishizuka Y Studies on the phosphorylation of myelin basic protein by protein kinase C and adenosine 3′:5′-monophosphate-dependent protein kinase. J Biol Chem 1985 260 12492 9 2413024

[38] Woodgett JR Gould KL Hunter T Substrate specificity of protein kinase C. Use of synthetic peptides corresponding to physiological sites as probes for substrate recognition requirements. Eur J Biochem 1986 161 177 84 3023081 10.1111/j.1432-1033.1986.tb10139.x

[39] Sheng X Zhang X Wu W Zhuang W Meng R Rong W Variants of RP1 gene in Chinese patients with autosomal dominant retinitis pigmentosa. Can J Ophthalmol 2008 43 208 12 18347624 10.3129/i08-004

